# The potential role of Arhgef33 RhoGEF in foveal development in the zebra finch retina

**DOI:** 10.1038/s41598-020-78452-6

**Published:** 2020-12-08

**Authors:** Takefumi Sugiyama, Haruka Yamamoto, Tetsuo Kon, Taro Chaya, Yoshihiro Omori, Yutaka Suzuki, Kentaro Abe, Dai Watanabe, Takahisa Furukawa

**Affiliations:** 1 Laboratory for Molecular and Developmental Biology, Institute for Protein Research, Osaka University, 3-2 Yamadaoka, Suita, Osaka 565-0871 Japan; 2 Department of Computational Biology and Medical Sciences, Graduate School of Frontier Sciences, The University of Tokyo, Chiba, 277-8562 Japan; 3 Laboratory of Brain Development, Graduate School of Life Sciences, Tohoku University, Miyagi, 980-8577 Japan; 4 Department of Biological Sciences, Graduate School of Medicine, Kyoto University, Kyoto, 606-8501 Japan

**Keywords:** Developmental biology, Neuroscience

## Abstract

The fovea is a pit formed in the center of the retina that enables high-acuity vision in certain vertebrate species. While formation of the fovea fascinates many researchers, the molecular mechanisms underlying foveal development are poorly understood. In the current study, we histologically investigated foveal development in zebra finch (*Taeniopygia guttata*) and found that foveal pit formation begins just before post-hatch day 14 (P14). We next performed RNA-seq analysis to compare gene expression profiles between the central (foveal and parafoveal) and peripheral retina in zebra finch at P14. We found that the Arhgef33 expression is enriched in the middle layer of the inner nuclear layer at the parafovea, suggesting that Arhgef33 is dominantly expressed in Müller glial cells in the developing parafovea. We then performed a pull-down assay using Rhotekin-RBD and observed GEF activity of Arhgef33 against RhoA. We found that overexpression of Arhgef33 in HEK293 cells induces cell contraction and that Arhgef33 expression inhibits neurite extension in Neuro 2A cells, which is partially recovered by a Rho-kinase (ROCK) inhibitor. Taken together, we used zebra finch as a model animal to investigate foveal development and identified Arhgef33 as a candidate protein possibly involved in foveal development through modulating RhoA activity.

## Introduction

High acuity of human vision largely depends on the fovea, which is a ‘pit’ structure formed at the center of the macula in the retina. The fovea is specialized to enhance the light path to photoreceptor cells^1^. Photoreceptor cell types include cones and rods responsible for photopic and scotopic vision, respectively. While the density of cone photoreceptor cells is highest in the fovea, the density of rod photoreceptor cells is almost zero in the fovea. In addition, the fovea projects to a larger fraction of the primary visual cortex than a comparable region of the periphery^2^. These features enable the fovea to be a high-acuity area in the retina. Retinal degenerative diseases affecting the fovea, including age-related macular degeneration and Stargardt’s disease, lead to vision loss. Histological analyses of the fovea during retinal development have been reported in human and monkey^1,3^. Moreover, gene expression profiles of the macula have been assessed using serial analysis of gene expression (SAGE) and RNA-sequencing^4,5^. Recently, the comparative analysis of RNAs isolated from the fovea and the periphery in the adult macaque retina using single-cell RNA sequencing revealed differences in gene expression profiles between these two regions^6^. However, little is known about the molecular basis underlying foveal development in the developing retina.

The fovea is formed in the retinas of limited number of species, including primates, birds, reptiles, and fish. Birds rely on their high visual acuity. Unlike primates, the number of foveae in the retina differs between birds. Many raptors, such as common buzzard and peregrine falcon, possess two foveae, whereas Canada goose and house sparrow develop a single fovea^7,8^. Among the birds, chickens are commonly used in experiments, but chicken has only relatively high-acuity area in the retina without a foveal pit. Regulation of retinoic acid (RA) signaling is involved in the formation of the high-acuity area in the chick retina^9^. Partly because of the lack of the sequenced genome data for foveate birds, analyses of foveal development using molecular biology in foveate birds are still insufficient.

Zebra finch (*Taeniopygia guttata*), a species of songbird, is one of the foveate birds and develops a single fovea in the retina. Recently, the genome of zebra finch has become available^10^. Zebra finch becomes the second bird (next to chicken) with sequenced whole genome. The visual functions of zebra finches were reported in terms of visual fields and the retinotopic map^11,12^. In addition, retinal histogenesis of zebra finch during the embryonic stages has recently been reported^13^. However, foveal development in zebra finch retina remains unknown.

In the present study, we examined foveal development using the zebra finch retina at several developmental stages. We performed comparative analysis of gene expression profiles between the central and peripheral retina in the developing zebra finch retina at the stage when the fovea is developing. We identified a candidate gene, *Arhgef33*, that encodes a small GTPase guanine nucleotide exchange factor (GEF) and shows differential expression between the central and peripheral retina. We found that Arhgef33 activates RhoA in cultured cells, suggesting that Arhgef33 may be involved in foveal development through the modulation of RhoA function.

## Results

### A mature fovea in the zebra finch retina

**Figure 1:**
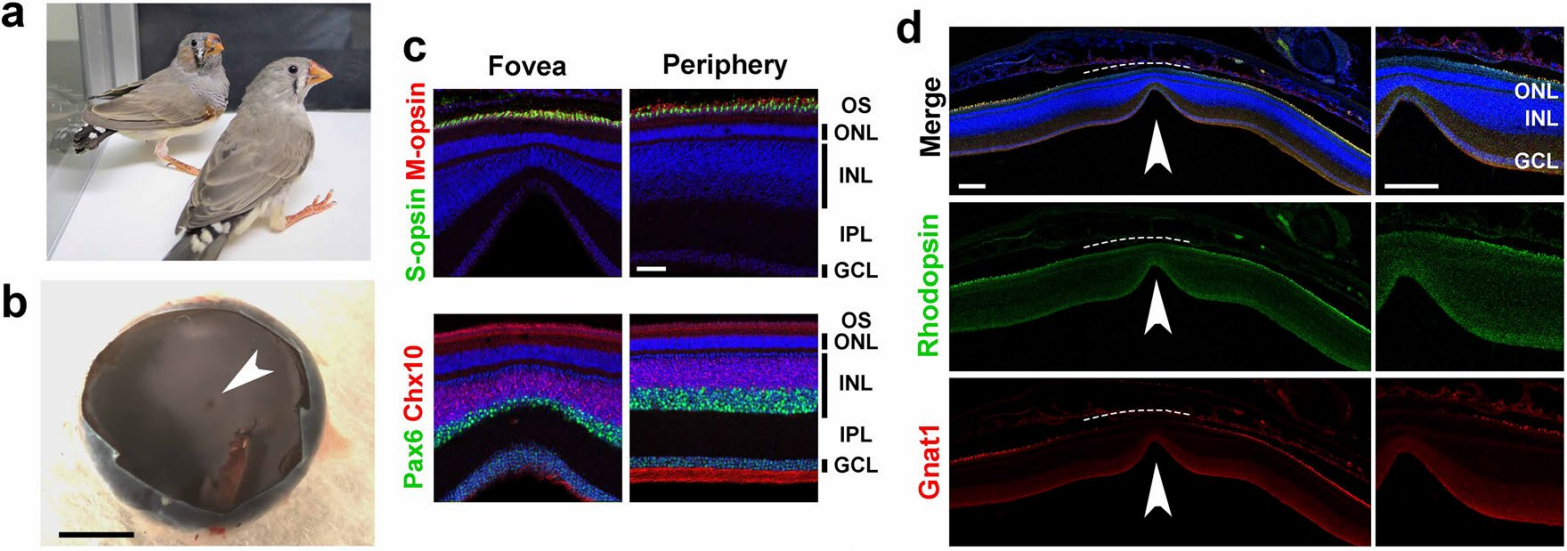
Immunostaining of the adult zebra finch fovea. (**a**) An image of zebra finches at P40. (**b**) A stereoscopic image of an adult zebra finch eye cup after removing the anterior part of the eye and vitreous body. The arrowhead indicates the fovea. Scale bar = 2 mm. (**c,d**) Immunostaining of retinal sections from adult zebra finches. (**c**) Retinal sections were stained with the anti-S-opsin (green) and anti-M-opsin (red) antibodies (upper panel), and the anti-Pax6 (green) and anti-Chx10 (red) antibodies (lower panel). Cell nuclei were stained with DAPI (blue). Cone photoreceptor, amacrine, ganglion and bipolar cells were present in both the fovea and the periphery in the retina. Scale bar = 50 μm. (**d**) Retinal sections were stained using anti-Rhodopsin (green) and anti-Gnat1 (red) antibodies, and DAPI (blue). Rod photoreceptor cells were present only in the periphery and absent in the fovea, indicating the existence of rod-free zone (dotted lines) in the zebra finch retina. The arrowhead indicates a foveal pit. Scale bar = 200 μm. OS, outer segment; ONL, outer nuclear layer; IPL, inner plexiform layer; INL, inner nuclear layer; GCL, ganglion cell layer.

In the current study we used retinas isolated from zebra finches (Fig. 1a). First, in order to examine the morphology and cellar composition of the adult zebra finch retina in detail, we performed immunostaining of retinal sections isolated from adult zebra finches at post-hatch day 200 (P200). The fovea of an adult zebra finch was present almost near the center of the eye cup (Fig. 1b). Cone opsins (S-opsin and M-opsin) were localized in the outer segment (OS) of photoreceptor cells. Signals of Pax6, a marker of amacrine and ganglion cells, and Chx10, a marker of bipolar cells, were observed in the inner nuclear layer (INL) (Fig. 1c). Pax6- and Chx10-positive cell layers were thinner in the foveal region than those in the peripheral region. We also observed rod OS markers, Rhodopsin and Gnat1*,* in the periphery of the retina, in contrast, did not observe these markers in the foveal region (Fig. 1d). These observations suggest that rod photoreceptor cells are absent, forming a rod-free zone in the adult zebra finch retina.

### Development of the fovea in the zebra finch retina

**Figure 2:**
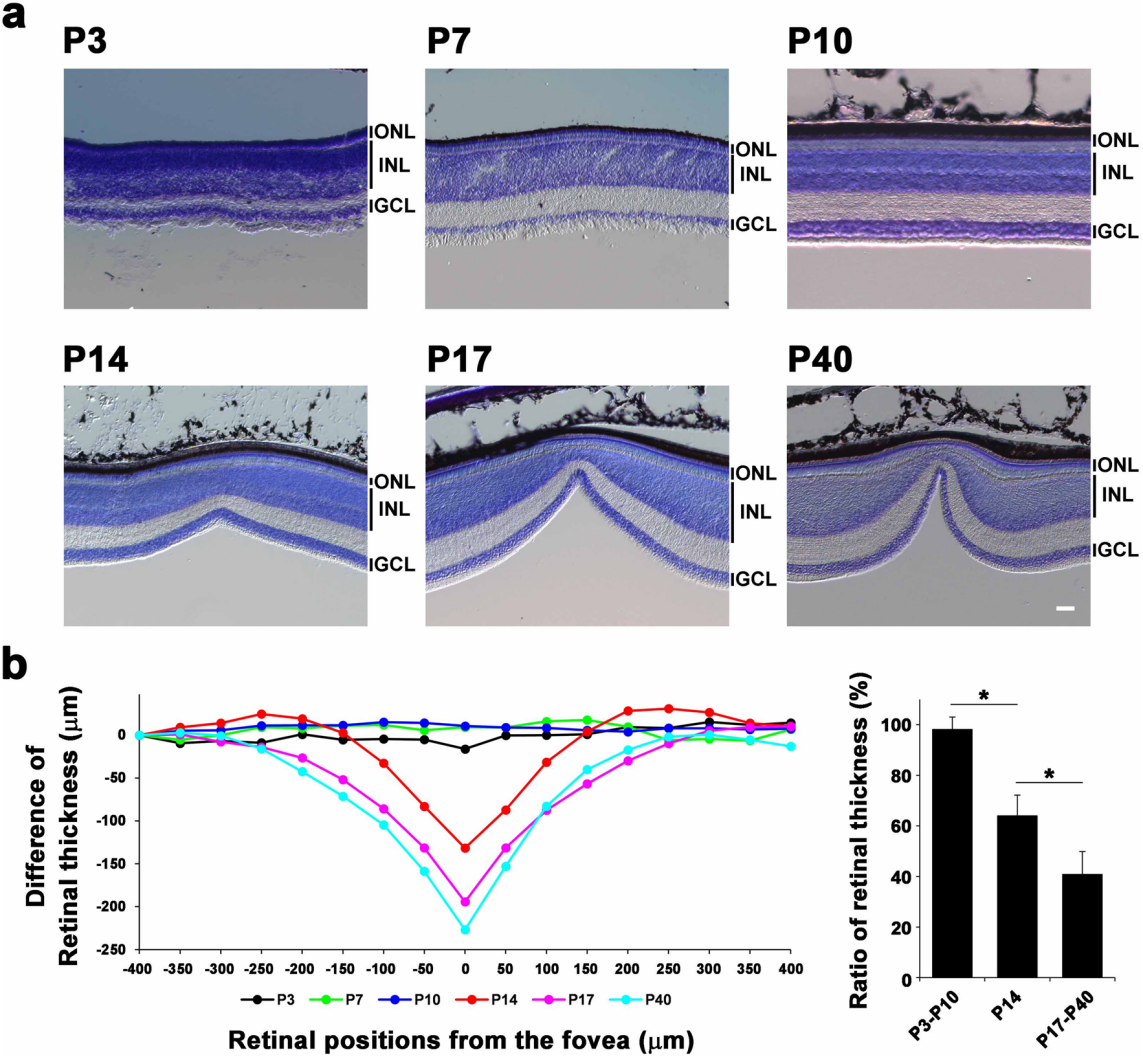
Foveal development in the zebra finch retina. (**a**) Toluidine blue staining of zebra finch retinal sections at P3, P7, P10, P14, P17, and P40. A foveal pit was not observed at P3, P7, or P10, but was detected at P14, P17, and P40. The depth of the fovea was deepened continuously from P14 to P40. Scale bar = 50 μm. ONL, outer nuclear layer; INL, inner nuclear layer; GCL, ganglion cell layer. (**b**) The representative differences in the thickness at each retinal position (at evenly spaced 50 μm intervals from nasal (− 400 μm) to temporal (400 μm) retina; 0 μm indicates the center of the fovea). The thickness from the most nasal position (− 400 μm) of the zebra finch retina at each stage was plotted and traced (left panel). The ratio of retinal thickness at the center of the fovea (0 μm) to the most nasal position (− 400 μm) was plotted for each developmental stage (P3 to P10, P14, and P17 to P40) (right panel). Significant differences assessed using a Student’s t-test are marked by an asterisk. One bird at P3, two birds at P7, one bird at P10, three birds at P14, two birds at P17, and two birds at P40 were used to examine one retina of each bird.

To investigate foveal development of zebra finches, we stained retinal sections of zebra finches at P3, P7, P10, P14, P17, and P40 with toluidine blue (Fig. 2a). The foveal pit was undetectable in the retina at P3, P7 and P10. We observed a shallow foveal pit in the retina at P14 and a deep foveal pit in the retina at P17 and P40. The three nuclear layers (ganglion cell layer, INL, and outer nuclear layer (ONL)) were observed at all developmental stages examined. To compare the depth of the fovea at each developmental stage, we measured retinal thickness at several retinal positions, at evenly spaced 50 μm intervals, in the retina and calculated the difference of the retinal thickness at each developmental stage (Fig. 2b, left panel). The ratio of the retinal thickness in the fovea to the periphery at P14 was significantly lower than that at P3–P10 and was significantly higher than that at P17–P40 (Fig. 2b, right panel). These results suggest that foveal formation in the zebra finch retina begins between P10 and P14.

### Gene expression profile in the developing fovea

**Figure 3:**
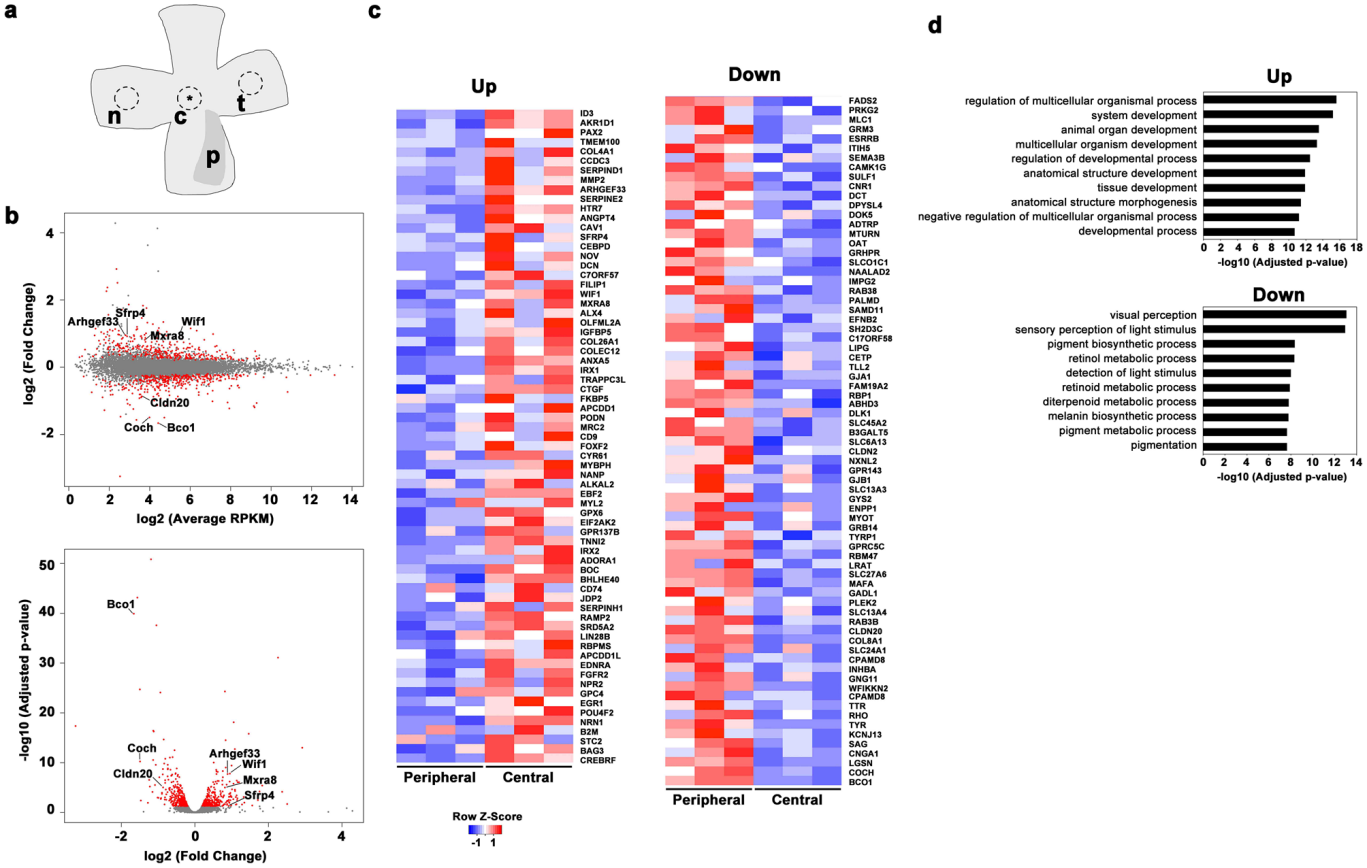
Expression profiles in the fovea. (**a**) A schematic depicting the areas where the punches were collected for RNA-seq analysis. The punch size was 1.5 mm in diameter. A fovea is marked by an asterisk. c, central; p, pecten; n, nasal; t, temporal. (**b**) The differences in gene expression profiles in between the fovea and the periphery are displayed in a MA plot (upper panel) and a volcano plot (lower panel). In the MA plot, M is log2 (FC), and A is log2 (average RPKM). In the volcano plot, X-axis denotes log2 (FC) and Y-axis denotes − log10 (adjusted p-value). Red color indicates genes with significant adjusted p-values (< 0.05). (**c**) A heat map of the genes with significant alterations in expression between the central and peripheral retina for the up-regulated and down-regulated genes (right and left panels). The peripheral retina contains both nasal and temporal retinal regions. Gene expression values were z-transformed for visualization using color scale from blue to red. (**d**) The top 10 most significantly enriched biological processes determined by gene ontology enrichment analysis for up-regulated and down-regulated genes (upper and lower panels). X-axis is − log10 (adjusted p-value) and Y-axis is biological processes.

Since we hypothesized that genes involved in foveal formation were likely to be expressed in the retina at P14, a time that we identified the fovea was in development, we decided to use the P14 zebra finch retina for gene expression analysis. In order to identify a gene(s) that regulates foveal development in zebra finches, we first performed RNA-seq analysis using total RNAs purified from the central and peripheral (nasal and temporal) retina at P14 zebra finch (Fig. 3a). After quality control, we identified 11,496 transcripts for both the central and peripheral retina in this analysis. We calculated the fold change (FC) in reads per kilobase of exon per million mapped sequence reads (RPKM) and plotted the relation of log2 (FC) to log2 (average RPKM) in the MA-plot (Fig. 3b; upper panel) and to -log10 (adjusted p-value) in the volcano plot (Fig. 3b; lower panel). Using the cut off (|log2 (FC) |> 0.5, log2 (RPKM) > 5, and FDR < 0.05), 69 genes were significantly up-regulated and 73 genes were significantly down-regulated in the central retina, as compared with those in the periphery (Fig. 3c). We then performed gene ontology (GO) analysis using PANTHER Classification System^14^. Up-regulated and down-regulated genes were classified into functional categories according to the GO term enrichment for biological processes. The top 10 significantly enriched biological processes were shown as for up-regulated and down-regulated genes (Fig. 3d). In the up-regulated genes, the genes involved in developmental processes, including tissue development and anatomical structure development, are enriched.

### Arhgef33 is enriched in the parafovea in the zebra finch retina

**Figure 4:**
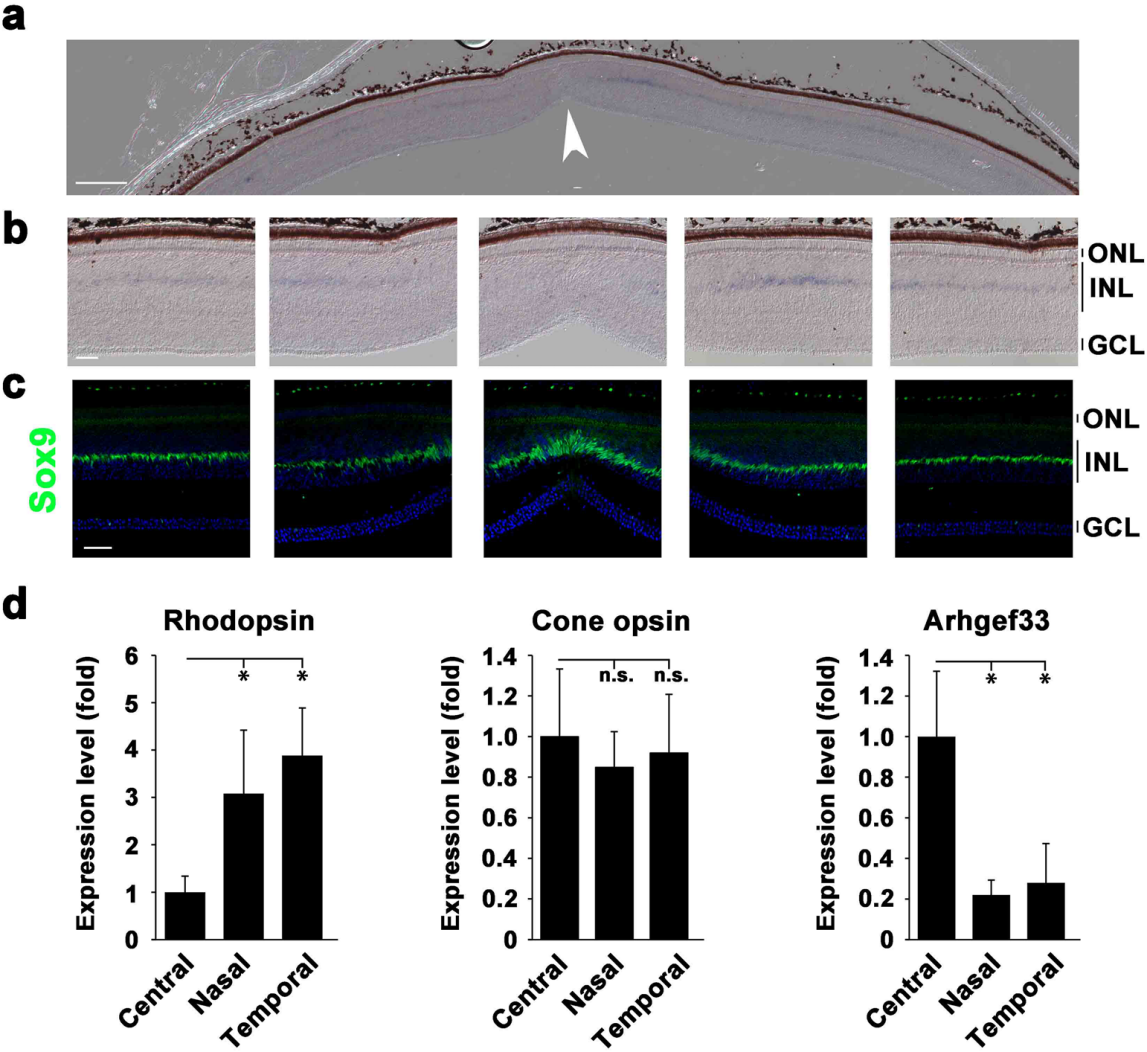
Specific expression of *Arhgef33* in the parafovea of the P14 zebra finch retina. (**a**) In situ hybridization analysis of *Arhgef33* expression in the zebra finch retina at P14. The arrowhead indicates a foveal pit. Scale bar = 200 μm. (**b**) Higher magnification images of the fovea (center) and parafovea (left, left center, right center, right). The *Arhgef33* expression was observed in the middle layer of the INL in the parafovea, while the *Arhgef33* expression was absent at the center of the fovea. Scale bar = 50 μm. ONL, outer nuclear layer; INL, inner nuclear layer; GCL, ganglion cell layer. (**c**) Immunostaining of P14 zebra finch retinal sections using an anti-Sox9 antibody (green) and DAPI (blue). Sox9 signal was detected in the middle layer of the INL. Scale bar = 50 μm. (**d**) Expression levels of *Rhodopsin* (left panel), *Cone opsin* (middle panel), and *Arhgef33* (right panel) were measured by RT-qPCR using RNAs purified from the foveal, nasal, and temporal regions of P14 zebra finch retinas. *Rhodopsin* expression was up-regulated in the nasal and temporal regions, while *Cone opsin* expression levels were not significantly different between each region. *Arhgef33* expression was up-regulated in the foveal region. Significant differences assessed using a Mann–Whitney U test are marked by an asterisk (n = 5; one retina of each bird). n.s., not significant.

Cell morphological changes in the fovea were reported based upon the histological observations seen in the developing fovea^3^. For the 142 genes that were up-regulated or down-regulated, we examined their reported or predicted gene functions in databases, and selected genes that have functions in biological processes, including cell adhesion, cell morphology, and cell migration, as suggested by the GO analysis. We selected seven candidate genes (Fig. 3b) and performed in situ hybridization analysis for these genes using the zebra finch retina at P14. Ultimately, we focused on Arhgef33 in the present study, because in situ hybridization showed enriched expression of *Arhgef33* in the central retina, whose function may regulate cell morphology and/or migration through small GTPases activity. We found that the *Arhgef33* expression in the central retina shows approximately a 2.1-fold up-regulation compared with that in the periphery (approximate FDR = 1.6 × 10^−8^). By in situ hybridization analysis, *Arhgef33* expression was observed to be restricted to the middle layer of the INL in the parafovea in the zebra finch retina at P14 (Fig. 4a,b, Fig. S1a), while the *Arhgef33* expression was not detected at the very center of the fovea. The cell bodies of Müller glial cells are known to be present in the middle portion of the INL in the chick retina^15^. The expression of *Sox9*, a marker of Müller glial cells, was prominent in the central retina and was present in the middle portion of the INL. The staining pattern of *Arhgef33* was similar to that of *Sox9* expression in the INL (Fig. 4c).

The expression levels of *Rhodopsin*, *Cone opsin*, and *Arhgef33* were quantified by reverse-transcription quantitative PCR (RT-qPCR) (Fig. 4d) using RNAs purified from the central, nasal, and temporal retina of P14 zebra finch retinas. Expectedly, the *Rhodopsin* expression levels were significantly higher in the periphery than that in the central retina at P14 (Fig. 4d; left panel), while the *Cone opsin* expression levels did not show a significant difference between the central and peripheral retina (Fig. 4d; middle panel). We found that the expression level of *Arhgef33* is significantly higher in the central retina than in the peripheral retina (Fig. 4d; right panel). As far as we examined *Arhgef33* expression in the adult retina by in situ hybridization, we did not observe a significant signal for *Arhgef33* (Fig. S1b), suggesting that *Arhgef33* is expressed only in the parafovea of the retina during development.

### Arhgef33 is a novel RhoA GEF regulating cell morphology

**Figure 5:**
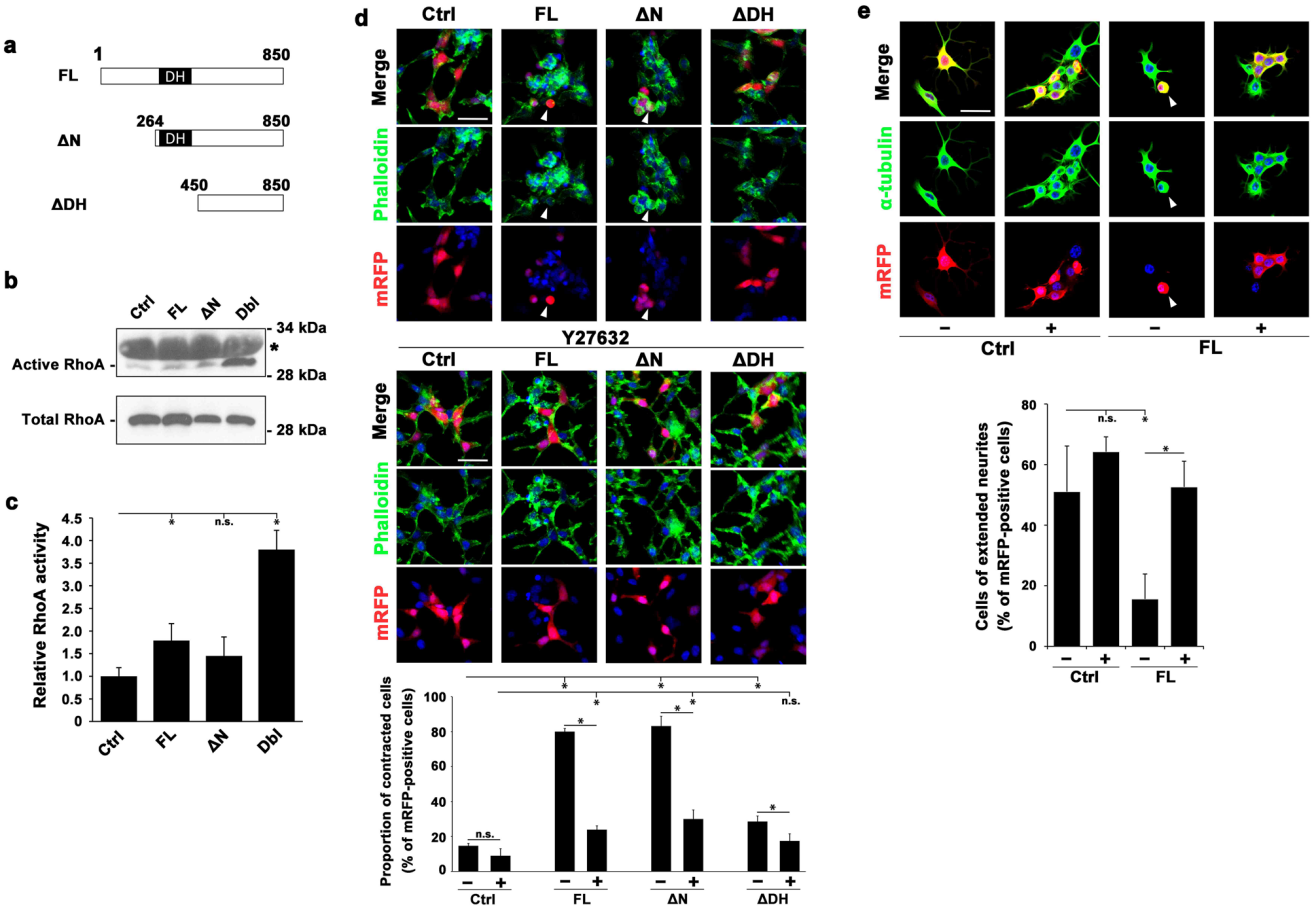
Arhgef33 exhibits a RhoA GEF activity. (**a**) Domain structures of Arhgef33. Full-length (FL, residues 1–850), N-terminal truncated (ΔN, residues 264–850) and DH-domain truncated (ΔDH, residues 450–850) Arhgef33 are illustrated. (**b,c**) The representative image of western blot analysis. (**b**) Bands of active (upper panel) and total (lower panel) HA-tagged RhoA in cell lysates from HEK293 cells transfected with plasmids coding genes depicted in each column were determined by immunoblotting with anti-HA antibody. Asterisk indicates non-specific bands. (**c**) Relative RhoA activities are indicated by the amount of active RhoA normalized to total RhoA. Significant differences assessed using a Student’s t-test are marked by an asterisk (three independent experiments). n.s., not significant. The full image of the western blots was shown in Fig. S1. (**d**) Immunofluorescence image of HEK293 cells co-transfected with plasmids encoding mRFP and each one of genes depicted in a column with/without treatment of Y27632 (upper and middle panels). The percentage of contracted cells relative to the total number of mRFP-positive cells (total cell number = 100 to 150) is shown (lower panel). Significant differences were assessed using a Student’s t-test are marked with an asterisk (three independent experiments). n.s., not significant. Scale bar = 50 μm. (**e**) Immunofluorescence image of RA-treated N2A cells co-transfected with plasmids encoding mRFP and an Arhgef33 or control plasmid with/without Y27632 (upper panel). The percentages of the number of cells with extended neurites against total number of mRFP-positive cells (total cell number = 30) were calculated (lower panel). Significant differences were assessed using a Student’s t-test are marked with an asterisk (three independent experiments). n.s., not significant. Scale bar = 50 μm.

RhoGEFs, e.g*.* Dbl and Dock, are responsible for the activation of Rho family small GTPases, including RhoA, Rac1 and Cdc42^16^. A functionally uncharacterized protein, Arhgef33, contains the Dbl-homology (DH) domain that was predicted to exhibit Rho-GEF activity^17^. We cloned the full-length Arhgef33 (Arhgef33FL) and generated an N-terminal truncation mutant (Arhgef33ΔN) of Arhgef33 that lacks 1-263 amino acid residues in the region of the N-terminal side to the DH domain, as well as a DH-domain truncation mutant (Arhgef33ΔDH) that lacks 1-449 amino acid residues, including the DH domain (Fig. 5a). In previous studies, the deletion of amino acid residues in the N-terminal side of the DH domain resulted in a constitutively active Rho-GEF, while the deletion of the DH domain resulted in the loss of function of the Rho-GEF^18,19^. Therefore, Arhgef33ΔN is expected to be constitutively active, while Arhgef33ΔDH is expected to have a deficiency in Rho-GEF activity.

Among small GTPases, RhoA is known to be associated with cellular contraction^20^. Therefore, we decided to focus on RhoA in the present study. To examine whether Arhgef33 possesses a GEF activity against RhoA, we performed a pull-down assay using the GST-tagged Rhotekin-RBD (Rho-binding domain)^21^. In the pull-down assay, cell lysates were mixed with GST-tagged Rhotekin-RBD beads, to which only active RhoA bound. We then measured the amount of HA-tagged RhoA in cell lysates (total RhoA) and the amount of HA-tagged RhoA bound to the beads (active RhoA). The amount of active RhoA significantly increased in the lysates isolated from the HEK293 cells expressing Arhgef33FL and Dbl than from the HEK293 cells expressing a control vector (Fig. 5b,c).

Rho family small GTPases are known to control cell morphology via several signaling cascades, including the Rho/ROCK signaling pathway^22,23^. Then, we examined cell morphological changes possibly induced by the Arhgef33 signaling pathway through RhoA activation using HEK293 and Neuro 2A (N2A) cells. Cell contraction of HEK293 cells induced by RhoA was previously reported^24^. We examined cell contraction induced by Arhgef33 with or without Y27632 treatment, an inhibitor of ROCKs (Fig. 5d, upper and middle panel). ROCKs phosphorylate various substrates, including the myosin light chain, which mediates cell migration and controls cell morphology through the formation of actin stress fibers^25^. To characterize cell contraction, HEK293 cells co-expressing the mRFP plasmid and each one of the experimental plasmids, either the control, Arhgef33FL, Arhgef33ΔN, or Arhgef33ΔDH plasmid, were co-stained with phalloidin to label F-actin, anti-DS-RED antibody for mRFP, and DAPI (4′,6-diamidino-2-phenylindole) to label DNA. We calculated the ratio of the number of contracted cells against total number of mRFP-positive cells (Fig. 5d, lower panel). We found that HEK293 cells expressing Arhgef33FL or Arhgef33ΔN without Y27632 showed significant cell contraction compared with the cells expressing a control vector without Y27632. Furthermore, the percentage of contracted cells induced by Arhgef33FL or Arhgef33ΔN was largely reverted with Y27632. Without treatment of Y27632, Arhgef33ΔDH displayed significant differences in the percentage of contracted cells compared with Arhgef33FL and Arhgef33ΔN, whereas Arhgef33ΔDH showed a significant but slightly higher percentage of contracted cells than the control.

We next examined the effect of Arhgef33 activity on neurite outgrowth of N2A cells after retinoic acid (RA) induction with or without Y27632 (Fig. 5e, upper panel). N2A cells are known to differentiate and develop elongated neurites with RA treatment^26^. A previous study showed that neurite outgrowth of RA-induced N2A cells is inhibited by RhoA activation by Arhgef1, a Rho-GEF^27^. In order to characterize neurite outgrowth, N2A cells co-expressing the mRFP plasmid and either the control or Arhgef33FL plasmid were co-stained with an anti-α-tubulin antibody, DS-RED, and DAPI. We assessed neurites, which were visualized by α-tubulin staining, and defined cells with more than one neurite with a length over 20 μm as cells with extended neurites. We calculated the ratio of the number of cells with extended neurites to the total number of mRFP-positive cells (Fig. 5e, lower panel). We observed that after RA treatment without Y27632, neurite outgrowth of N2A cells expressing Arhgef33FL was significantly inhibited in contrast to that of N2A cells expressing a control vector. Conversely, neurite outgrowth of N2A cells expressing Arhgef33FL after RA treatment was significantly recovered with Y27632 treatment, suggesting that Arhgef33 activates the RhoA/ROCK signaling pathway, leading to induction of cell morphological changes of N2A cells.

## Discussion

### Zebra finches are an excellent model animal to study foveal development

It is likely that the basic molecular mechanisms underlying foveal development are conserved among species, because basic retinal structures and the foveal pit formation timing which begins after the completion of retinal layer differentiation are conserved^1, 3^. In the current study, we used zebra finch as a model animal to investigate foveal development. We observed that the foveal pit in zebra finch retina begins formation between P10 and P14. Since foveal pit formation was reported to begin before 24–26 weeks of gestation in the human embryonic retina^28^, a relatively delayed foveal pit formation after birth may be a useful characteristic of the zebra finch. This delay in the timing of foveal pit formation may associated with the shorter gestation time of the zebra finch than that of humans and chickens. While zebra finch takes only 14 embryonic days until hatch, human takes approximately 40 weeks of gestation and chicken takes 21 embryonic days^29^. In this aspect, to study foveal development in the zebra finch retina can be an approachable model for experimental procedures. The green anole (*Anolis carolinensis*) could be another model animal used to study foveal development, because several features of the fovea in *Anolis carolinensis*, such as increased density of cone photoreceptor cells and elongation of photoreceptor OSs, are similar to those of the human fovea^30^. However, foveal pit formation in *Anolis carolinensis* was reported to begin at embryonic stage 17^30^, which may accompany difficulties in experimental manipulation.

The mature zebra finch fovea has several features distinct from those in humans. Similar to other foveated birds, zebra finches have a funnel-shaped fovea, known as convexiclivate fovea, which differs from the dish- or bowl-shaped fovea present in humans^31^. It was reported that almost all of INL cells in the human fovea relocate from the center of the fovea to the periphery during development^3^. In contrast, INL cells are present in the center of the fovea even in the adult zebra finch retina. Thus, there are several differences in the mature fovea between human and zebra finch. However, since these differences appear at the later phases of foveal development, it is likely that the molecular mechanisms underlying the initial foveal pit formation are conserved among species. Even if the molecular mechanisms of foveal development are not conserved among species, including humans, birds, and reptiles, the current study may still shed light on the molecular mechanisms of foveal development in birds. Comparison of the molecular bases of foveal development among species awaits future studies.

### Differential expression of Arhgef33 in foveal development

In the present study, we investigated the gene expression profiles of the zebra finch fovea at the stage of foveal pit formation. We identified 69 up-regulated and 73 down-regulated genes in the central retina as compared with those in the periphery. A recent study identified cell type-specific gene expression profiles in human and monkey retinas using single-cell RNA sequencing data^6^. However, this study examined a mature retina, not a developing retina. The retinal transcriptome of the human retina at several developmental stages (including fetal day 132, which is just before foveal pit formation) was previously reported, but due to the small sample size it is difficult to investigate foveal development using these data^32^. Our current study revealed differences in the gene expression profiles between the central retina and the periphery soon after the foveal pit begins to form. Our RNA-seq data may be useful to analyze gene expression profiles not only for zebra finch but also for other foveated species, including human and macaque.

By comparing the gene expression profiles between the central retina and the periphery in the developing zebra finch retina, we identified several genes whose expression is enriched in the central retina. We examined their expression patterns in the retina using in situ hybridization on developing zebra finch retinal sections, and found that the expression of Arhgef33, a functionally unknown GEF-coding gene, is enriched at the parafovea in the zebra finch retina at P14. *Arhgef33* expression was observed in the middle layer of the INL, which mainly contains four types of cells; amacrine cells, bipolar cells, horizontal cells, and Müller glial cells^33^. In the vertebrate retina, amacrine cells localize to the inner parts of the INL, and bipolar and horizontal cells localize to the outer parts of the INL, whereas Müller glial cells are localized in the middle layer of the INL. To confirm the localization of cell bodies of Müller glial cells in the zebra finch INL, we immunostained the retina with a Sox9 antibody, a marker for Müller glial cells. We observed that Sox9 signals are localized to the middle layer of the INL, indicating that Müller glial cells are localized in the middle layer of the INL in the zebra finch retina. Localization of the Sox9-positive cells was very similar to the Arhgef33 expression pattern in the middle layer of the INL. These results suggest that Arhgef33 is very likely to be expressed in Müller glial cells in the parafovea of the developing zebra finch retina. We used antibodies that were not verified for zebra finches in the current study; therefore, immunostaining of the zebra finch retina using such antibodies should be carefully assessed. The localizations of immunostained signals observed in the current study recapitulate the immunostaining patterns in mice. However, the verification of other antibodies across species may be necessary in future studies.

### Possible involvement of Arhgef33 in foveal development

We examined the RhoA-GEF activity of Arhgef33 using a pull-down assay with Rhotekin-RBD and a cell morphological assay in cultured cells. Rhotekin-RBD exhibits binding activity to RhoA, RhoB, and RhoC^21^. Arhgef10, which has RhoA-GEF activity, was previously shown to result in cell contraction of HEK293 cells when overexpressed^24^. Neurite outgrowth of N2A cells was negatively regulated by Arhgef1 through the RhoA signaling pathway^27^. Similarly, Arhgef33 expression induced cell contraction of HEK293 cells and inhibited neurite outgrowth of N2A cells, suggesting that Arhgef33 contains RhoA-GEF activity. These data suggest that the RhoA-GEF activity of Arhgef33 depends on the DH domain. The RhoA-GEF activity of Arhgef33 is less than that of Dbl, but the activity is enough to induce the cell morphological changes. Arhgef33ΔN did not have statistically significant RhoA activation activity. However, Arhgef33ΔN exhibited a tendency to activate RhoA. We hypothesize that the modestly increased RhoA activation activity of Arhgef33ΔN may be enough to show cell contraction activity in cultured cells. Indeed, the cell contraction induced by Arhgef33FL and Arhgef33ΔN was largely reverted with treatment of a ROCK inhibitor, Y27632. However, the percentage of the contracted cells induced by Arhgef33FL and Arhgef33ΔN were still higher than that of the control with treatment of the ROCK inhibitor, which suggests that ROCK-independent pathway(s) are involved in the cell contraction induced by Arhgef33. Unexpectedly, Arhgef33ΔDH induced cell contraction more frequently than the control, although the percentage of contracted cells induced by Arhgef33ΔDH was substantially less than that induced by Arhgef33FL and Arhgef33ΔN. This suggested that the C-terminal side to the DH domain in Arhgef33 possesses weak Rho-GEF activity.

We also found that the RhoA-ROCK signaling pathway is activated by Arhgef33. The ROCK signaling pathway was reported to be responsible for the phenotypic change of Müller glial cells under hypoxia or oxidative stress^34^. Although the expression of small GTPases was examined in the developing vertebrate retina^35,36^, the role of the Rho/ROCK signaling pathway in Müller glial cells during the retinal development is not well understood. Regarding other glial cells, it was reported that RhoA inhibits the branching of oligodendrocytes and negatively regulates process growth and migration of astrocytes after injury^37,38^.

The contribution of Müller glial cells to foveal pit formation has been proposed previously^39^. The cooperation of Müller glial cells with astrocytes may cause the vertical contraction of Müller glial cells, resulting in foveal pit formation^31^. Although we could not obtain direct evidence of *Arhgef33* expression in Müller glial cells in the current study, we propose that a molecular mechanism is responsible for foveal development: morphological changes in Müller glial cells in the parafoveal region are induced by Arhgef33 through the Rho/ROCK signaling pathway, leading to foveal pit formation. Future in vivo studies are needed to test this hypothetical model by overexpression and/or targeted gene disruption/knockdown of *Arhgef33* in the zebra finch retina.

## Experimental procedures

### Animal care

All experiments were approved by the Animal Care and Use Committee of Kyoto University. All experiments and methods were performed in accordance with relevant guidelines and regulations. Zebra finches used in this study were bred and kept in the aviary at Kyoto University, according to the published guideline^40^. A single breeding pair was kept in a cage of 42 × 33 × 33 cm, in rooms with a 14 h light, 10 h dark period. Food and water were given ad libitum throughout the rearing and the analysis. During the breeding, the nests of zebra finch pairs were checked once a day to determine the hatch day for each bird. Juvenile birds were kept with their parents until P60. Afterward, they were moved to a new cage and kept with birds of same sex with similar age. Birds were euthanatized with ketamine–xylazine before tissue collection. In total, we used one bird at P3, two birds at P7, one bird at P10, eight birds at P14, two birds at P17, two birds at P40, and one bird at P200.

### Plasmid construction

In order to construct the plasmid expressing FLAG-tagged full-length Arhgef33 and FLAG-tagged Arhgef33 N-terminal or DH-domain deletion mutant, cDNA fragments encoding full length (residues 1–850), N-terminal truncated Arhgef33 (residues 264–850), and DH-domain truncated Arhgef33 (residues 450–850) were amplified from a mouse cerebellum cDNA library by PCR, and cloned into the pCAGGSII expression vector^41^. The full-length cDNA of mouse *RhoA* isolated from mouse retinal RNAs by RT-PCR was cloned into pCAGGSII containing 2 × HA tags. The pCAGGSII expression vector without insert was used for control.

### Preparation of the zebra finch retina

Zebra finch eye cups at P3, P7, P10, P14, P17, and P40, and the adult stage were fixed in 4% paraformaldehyde (PFA) in phosphate-buffered saline (PBS) for 30 min for immunofluorescent analysis, or overnight for toluidine blue staining and in situ hybridization. Cryosections were cut at a thickness of 20 µm.

### In situ hybridization

In situ hybridization was performed as described previously^41^. In our current study, we modified the proteinase K treatment time from 5 to 10 min. The cDNA fragments of zebra finch *Rhodopsin*, *S-opsin* (cone opsin) and *Arhgef33* were amplified by PCR from zebra finch retinal cDNA with the primers shown in Table S1. Digoxigenin-labeled riboprobes for those genes were prepared by in vitro transcription with 11-digoxigenin UTPs (Roche).

### qRT-PCR analysis

qRT-PCR analysis was performed as described previously^42^. For the retinal punches, 1.5-mm-diameter punches from the central, nasal, and temporal regions of the retina were punched out from three individual zebra finches at P14. The punches from each animal were pooled for each sample, and the RNA was isolated using Trizol reagent (Thermo Fisher Scientific). The cDNA was prepared using SuperScript II Reverse Transcriptase (Thermo Fisher Scientific). qRT-PCR was performed using SYBR Green ER qPCR Super Mix (Thermo Fisher Scientific) and Thermal Cycler Dice Real Time System Single MR Q TP870 (Takara Bio) according to the manufacturer’s instructions. Quantification was carried out using the Thermal Cycler Dice Real Time System software version 2.0 (Takara Bio). The primer sequences are shown in Table S1.

### Immunofluorescent analysis of retinal sections and cultured cells

Immunofluorescent analysis was performed as described previously^42^. Zebra finch eye cups were fixed with 4% PFA in PBS for 30 min at room temperature. The eye cups were rinsed in PBS and cryoprotected in 30% sucrose/PBS overnight at 4 °C. The samples were embedded in Tissue-Tek OCT compound 4583 (Sakura), frozen on dry ice, and sectioned (20 μm) using a MICROM HM560 cryostat (Thermo Fisher Scientific). Sections on slides were dried for 2 h at room temperature, rehydrated in PBS for 5 min, and incubated with the primary antibodies in blocking buffer (5% normal donkey serum and 0.1% Triton X-100 in PBS) at 4 °C overnight. The slides were washed with PBS, incubated with the secondary antibodies in blocking buffer for 2 h at room temperature and coverslipped with gelvatol after being washed with PBS. The specimens were observed under a laser confocal microscope (LSM700, Carl Zeiss).

HEK293 and N2A cells were cultured in DMEM (Sigma) with 10% fetal bovine serum (FBS). HEK293 and N2A cells were transfected using calcium phosphate buffer and Lipofectamine LTX (Thermo Fisher), respectively. After 48 h or 72 h of transfection, cells were fixed in 4% PFA/PBS for 10 min at room temperature. Immunofluorescent staining was performed as described for tissue sections.

We used the following primary antibodies: anti-Rhodopsin (1:2,500, LSL, LB-5597), anti-M-opsin (1:300, Millipore, AB5405), anti-S-opsin (1:500, Santa Cruz, sc-14363), anti-Pax6 (1:500; Developmental Studies Hybridoma Bank, PAX6), anti-Gnat1 (1:500, Santa Cruz, sc-389), anti-Chx10 (1:100, in house)^43^, anti-Sox9 (1:500, Millipore, AB5535), anti-α tubulin (1:500, Sigma, T9026), and anti-DS RED (1: 1,000, Clontech, 632,496) antibodies. Cy3-conjugated (1:500, Jackson ImmunoResearch Laboratories) or Alexa Fluor 488-conjugated secondary antibodies (1:500, Sigma) were used. AF488-conjugated Phalloidin (1:500, Invitrogen) was used for staining F-actin.

### GST-fused Rhotekin-RBD beads

GST-fused Rhotekin-RBD (GST-RBD) was expressed in *Escherichia coli* strain BL21 (DE3) transformed with pGEX-5X-1-Rhotekin-RBD (kindly provided by Drs. S. Inagaki and S. Shibata)^44^. After the treatment of 0.1 mM isopropyl β-D-thiogalactopyranoside for 4 h at room temperature, cells were harvested by centrifugation and lysed in the extraction buffer (20 mM Tris–HCl (pH 7.5), 1 mM EDTA, 150 mM NaCl, 0.1% TritonX-100, 2 mM DTT, 10 mM MgCl_2_, 0.1 mM PMSF). The supernatants were mixed with glutathione Sepharose 4B (GE Healthcare) for 1 h at 4 °C. Concentration of purified protein was quantified by SDS-PAGE and Coomassie Brilliant Blue staining.

### Pull down assay

HEK293 cells were cultured in DMEM (Sigma) with 10% FBS and co-transfected using the calcium phosphate method with plasmids encoding HA-tagged RhoA and either the control vector or plasmid encoding Flag-tagged Arhgef33 full length, N-terminal deletion mutant, or Dbl. Pull down assay was performed as described previously^24^. After 48 h of transfection, cells were washed with ice-cold Tris-buffered saline and lysed with lysis buffer (50 mM Tris–HCl (pH 7.5), 1% NP-40, 150 mM NaCl, 10 mM MgCl_2_, 1 mM EDTA, 10% glycerol, 25 mM NaF, 0.1 mM PMSF). The supernatants were mixed with GST-fused Rhotekin-RBD beads for 45 min at 4 °C. The beads were washed with the lysis buffer and the protein bound to the beads was eluted using the SDS-sample buffer. The protein was then subjected to SDS-PAGE and immunoblotting using an anti-HA antibody (3F10, 1:1000, Roche). The band intensity of the immunoblot was quantified using ImageJ software (National Institutes of Health, Bethesda, MD) and the amount of active RhoA was normalized to the total amount of RhoA.

### Cell contraction induction

HEK293 cells were cultured in DMEM (Sigma) with 10% FBS and co-transfected using the calcium phosphate method with plasmids encoding mRFP and either a control vector or plasmid encoding Arhgef33FL, Arhgef33ΔN, and Arhgef33ΔDH with/without 10 μM Y27632. After 24 h, the medium was changed to DMEM with 10% FBS with/without 10 μM Y27632. Cells were incubated for an additional 24 h and fixed in 4% PFA/PBS for 10 min at room temperature. Immunofluorescent staining was performed as described above.

### Cell differentiation induction

N2A cells were cultured in DMEM (Sigma) with 10% FBS and co-transfected using Lipofectamine LTX (Thermo Fisher Scientific) with plasmids encoding mRFP and either a control vector or plasmid encoding Arhgef33FL. After 12 h, the medium was changed to DMEM with 2% FBS, and 12 h after that, the medium was changed to DMEM with 2% FBS containing 20 μM retinoic acid (RA) with/without 10 μM Y27632. Cells were incubated for additional 48 h and fixed in 4% PFA/PBS for 10 min at room temperature. Immunofluorescent staining was performed as described above.

### Western blot analysis

Western blot analysis was performed as described previously^45^. Samples were resolved by SDS-PAGE, and transferred to polyvinylidene difluoride membranes (Millipore) using a semi-dry transfer cell (Bio-Rad). The membranes were blocked with a blocking buffer (total 0.1% Tween 20 and 4% skim milk in PBS) and incubated with primary antibodies in the blocking buffer overnight at 4 °C. The membranes were washed three times for 10 min with 0.1% Tween 20 in TBS, and incubated with secondary antibodies in the blocking buffer for 2 h at room temperature. Signals were detected using Chemi-Lumi One L (Nacalai, Japan) or Pierce Western Blotting Substrate Plus (Thermo Fisher Scientific). We used a rat monoclonal anti-HA (1:5,000, Roche, 3F10) antibody as the primary antibody and horseradish peroxidase-conjugated anti-mouse IgG (1:10,000, Zymed) antibody as the secondary antibody.

### RNA-seq and analysis of RNA-seq data

Dissected foveal and non-foveal retinal regions at P14 and adult stage were homogenized in Trizol reagent (Thermo Fisher Scientific) and stored at − 80 °C. Total RNAs were extracted following manufacturer’s instructions. Single-end sequencing was performed at a length of 36 bases using the HiSeq2500 (Illumina). A read length of 36 bases is considered long enough to map the reads to cover the whole genome^46^. The reference genome sequence of zebra finch (taeGut3.2.4) was downloaded from Ensembl database. After basic quality control, RNA-seq reads were aligned to the reference genome using Illumina ELAND v2, followed by quantification of read counts for each gene. The gene expression matrix was loaded on R-3.3.3. Differentially expressed genes (DEGs) were identified using edgeR^47^. Genes with |log2 (FC) |> 0.5, log2 (RPKM) > 5, and FDR < 0.05 were considered significantly changed. Heatmap of DEGs was visualized using heatmap.2 function in gplots library. To perform Gene Ontology (GO) analysis, 199 DEGs with orthologs in mice were analyzed. GO analysis was performed using PANTHER Classification System^14^.

### Statistical analysis

Statistical significance was calculated using a Student's t-test or Mann–Whitney U test after testing for normality with a Shapiro–Wilk test and deviations with F-test. A value of *p* < 0.05 was considered statistically significant. Data are presented as the mean ± SD.

## Supplementary information

Supplementary Information.

## Data Availability

RNA-seq reads can be obtained in DDBJ Sequence Read Archive (DRA) under accession number DRA010159.

## References

[1] Provis JM, Dubis AM, Maddess T, Carroll J (2013). Adaptation of the central retina for high acuity vision: cones, the fovea and the avascular zone. Prog. Retin. Eye Res..

[2] Wandell BA, Dumoulin SO, Brewer AA (2007). Visual field maps in human cortex. Neuron.

[3] Hendrickson AE, Yuodelis C (1984). The morphological development of the human fovea. Ophthalmology.

[4] Sharon D, Blackshaw S, Cepko CL, Dryja TP (2002). Profile of the genes expressed in the human peripheral retina, macula, and retinal pigment epithelium determined through serial analysis of gene expression (SAGE). Proc. Natl. Acad. Sci. USA.

[5] Li M (2014). Comprehensive analysis of gene expression in human retina and supporting tissues. Hum. Mol. Genet..

[6] Peng Y-R (2019). Molecular classification and comparative taxonomics of foveal and peripheral cells in primate retina. Cell.

[7] Mitkus M, Olsson P, Toomey MB, Corbo JC, Kelber A (2017). Specialized photoreceptor composition in the raptor fovea. J. Comp. Neurol..

[8] Moore BA, Tyrrell LP, Pita D, Bininda-Emonds ORP, Fernández-Juricic E (2017). Does retinal configuration make the head and eyes of foveate birds move?. Sci. Rep..

[9] da Silva S, Cepko CL (2017). Fgf8 expression and degradation of retinoic acid are required for patterning a high-acuity area in the retina. Dev. Cell.

[10] Warren WC (2010). The genome of a songbird. Nature.

[11] Bischof H-J (1988). The visual field and visually guided behavior in the zebra finch (*Taeniopygia guttata*). J. Comput. Physiol..

[12] Keary N, Voss J, Lehmann K, Bischof H-J, Löwel S (2010). Optical imaging of retinotopic maps in a small songbird, the zebra finch. PLoS ONE.

[13] Álvarez-Hernán G (2018). Retinal histogenesis in an altricial avian species, the zebra finch (*Taeniopygia guttata*, Vieillot 1817). J. Anat..

[14] Mi H (2019). Protocol Update for large-scale genome and gene function analysis with the PANTHER classification system (v140). Nat. Protocols.

[15] Xue LP (2006). Müller glial cells express nestin coupled with glial fibrillary acidic protein in experimentally induced glaucoma in the rat retina. Neuroscience.

[16] Bos JL, Rehmann H, Wittinghofer A (2007). GEFs and GAPs: critical elements in the control of small G proteins. Cell.

[17] Rossman KL, Der CJ, Sondek J (2005). GEF means go: turning on RHO GTPases with guanine nucleotide-exchange factors. Nat. Rev. Mol. Cell Biol..

[18] Bi F (2001). Autoinhibition mechanism of proto-Dbl. Mol. Cell Biol..

[19] Yu B (2010). Structural and energetic mechanisms of cooperative autoinhibition and activation of Vav1. Cell.

[20] Da Silva JS (2003). RhoA/ROCK regulation of neuritogenesis via profilin IIa-mediated control of actin stability. J. Cell Biol..

[21] Ren XD, Schwartz MA (2000). Determination of GTP loading on Rho. Meth. Enzymol..

[22] Nobes CD, Hall A (1995). Rho, rac, and cdc42 GTPases regulate the assembly of multimolecular focal complexes associated with actin stress fibers, lamellipodia, and filopodia. Cell.

[23] Nakagawa O (1996). ROCK-I and ROCK-II, two isoforms of Rho-associated coiled-coil forming protein serine/threonine kinase in mice. FEBS Lett..

[24] Chaya T (2011). Identification of a negative regulatory region for the exchange activity and characterization of T332I mutant of Rho guanine nucleotide exchange factor 10 (ARHGEF10). J. Biol. Chem..

[25] Riento K, Ridley AJ (2003). Rocks: multifunctional kinases in cell behaviour. Nat. Rev. Mol. Cell Biol..

[26] Riboni L, Prinetti A, Bassi R, Caminiti A, Tettamanti G (1995). A mediator role of ceramide in the regulation of neuroblastoma Neuro2a cell differentiation. J. Biol. Chem..

[27] Xiang X, Li S, Zhuang X, Shi L (2016). Arhgef1 negatively regulates neurite outgrowth through activation of RhoA signaling pathways. FEBS Lett..

[28] Yuodelis C, Hendrickson A (1986). A qualitative and quantitative analysis of the human fovea during development. Vis. Res..

[29] Hamburger V, Hamilton HL (1992). A series of normal stages in the development of the chick embryo. 1951. Dev. Dyn..

[30] Sannan NS, Shan X, Gregory-Evans K, Kusumi K, Gregory-Evans CY (2018). Anolis carolinensis as a model to understand the molecular and cellular basis of foveal development. Exp. Eye Res..

[31] Bringmann A (2018). The primate fovea: structure, function and development. Prog. Retin. Eye Res..

[32] Hoshino A (2017). Molecular anatomy of the developing human retina. Dev. Cell.

[33] Dowling JE (1970). Organization of vertebrate retinas. Invest. Ophthalmol..

[34] Zhang X-H, Feng Z-H, Wang X-Y (2018). The ROCK pathway inhibitor Y-27632 mitigates hypoxia and oxidative stress-induced injury to retinal Müller cells. Neural Regen. Res..

[35] Mitchell DC (2007). Developmental expression of three small GTPases in the mouse eye. Mol. Vis..

[36] Santos-Bredariol AS, Belmonte MA, Kihara AH, Santos MF, Hamassaki DE (2006). Small GTP-binding protein RhoB is expressed in glial Müller cells in the vertebrate retina. J. Comput. Neurol..

[37] Avalos AM (2004). Aggregation of integrins and RhoA activation are required for Thy-1-induced morphological changes in astrocytes. J. Biol. Chem..

[38] Wang H (2012). Myosin II is a negative regulator of oligodendrocyte morphological differentiation. J. Neurosci. Res..

[39] Snyder AW, Miller WH (1978). Telephoto lens system of falconiform eyes. Nature.

[40] Olson CR, Wirthlin M, Lovell PV, Mello CV (2014). Proper care, husbandry, and breeding guidelines for the zebra finch, *Taeniopygia guttata*. Cold Spring Harb. Protoc..

[41] Yamamoto H, Kon T, Omori Y, Furukawa T (2020). Functional and evolutionary diversification of Otx2 and Crx in vertebrate retinal photoreceptor and bipolar cell development. Cell Rep..

[42] Omori Y (2017). Samd7 is a cell type-specific PRC1 component essential for establishing retinal rod photoreceptor identity. Proc. Natl. Acad. Sci. USA.

[43] Sanuki R (2011). miR-124a is required for hippocampal axogenesis and retinal cone survival through Lhx2 suppression. Nat. Neurosci..

[44] Yamada T, Ohoka Y, Kogo M, Inagaki S (2005). Physical and functional interactions of the lysophosphatidic acid receptors with PDZ domain-containing Rho guanine nucleotide exchange factors (RhoGEFs). J. Biol. Chem..

[45] Ueno A (2018). Lrit1, a retinal transmembrane protein, regulates selective synapse formation in cone photoreceptor cells and visual acuity. Cell Rep..

[46] Chhangawala S, Rudy G, Mason CE, Rosenfeld JA (2015). The impact of read length on quantification of differentially expressed genes and splice junction detection. Genome Biol..

[47] Robinson MD, McCarthy DJ, Smyth GK (2010). edgeR: a Bioconductor package for differential expression analysis of digital gene expression data. Bioinformatics.

