# Supplementary material for: The potential role of Arhgef33 RhoGEF in foveal development in the zebra finch retina
Source: Sci Rep. 2020 Dec 8;10:21450. doi: 10.1038/s41598-020-78452-6 (PMC7722920; doi:10.1038/s41598-020-78452-6)
Supplement: Supplementary file 1 — Supplementary Information. [file 41598_2020_78452_MOESM1_ESM.pdf]

Supplementary information

The potential role of Arhgef33 RhoGEF in foveal development in the zebra finch retina

Takefumi Sugiyama[1], Haruka Yamamoto[1], Tetsuo Kon[1], Taro Chaya[1], Yoshihiro Omori[1], Yutaka Suzuki[2], Kentaro Abe[3,4], Dai Watanabe[4], Takahisa Furukawa[1*]

[1]Laboratory for Molecular and Developmental Biology, Institute for Protein Research, Osaka University, Osaka, 565-0871, Japan
[2]Department of Computational Biology and Medical Sciences, Graduate School of Frontier Sciences, The University of Tokyo, Chiba, 277-8562, Japan
[3]Laboratory of Brain Development, Graduate School of Life Sciences, Tohoku University, Miyagi, 980-8577, Japan
[4]Department of Biological Sciences, Graduate School of Medicine, Kyoto University, Kyoto, 606-8501, Japan

[*]Corresponding author: Takahisa Furukawa

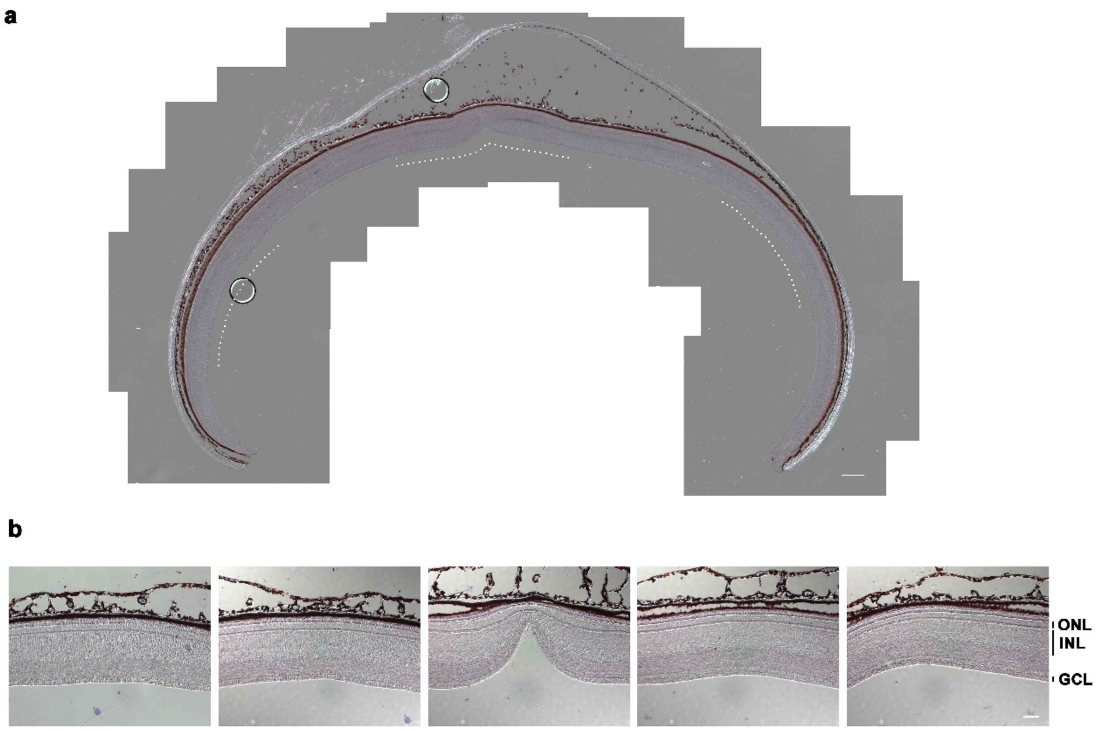

Figure S1. The Arhgef33 expression in the zebra finch retina at P14 and adult stage.

(**a**) The whole image of the retinal section. *In situ* hybridization analysis of Arhgef33 expression in the zebra finch retina at P14. Dotted lines indicate the approximate locations punched out for RNA-seq. Scale bar = 200 µm.

(**b**) Higher magnification images of the fovea (center) and parafovea (left, left center, right center, right). *In situ* hybridization analysis of Arhgef33 expression in the adult zebra finch retina. No *Arhgef33* signal was observed. Scale bar = 50 µm. ONL, outer nuclear layer; INL, inner nuclear layer; GCL, ganglion cell layer.

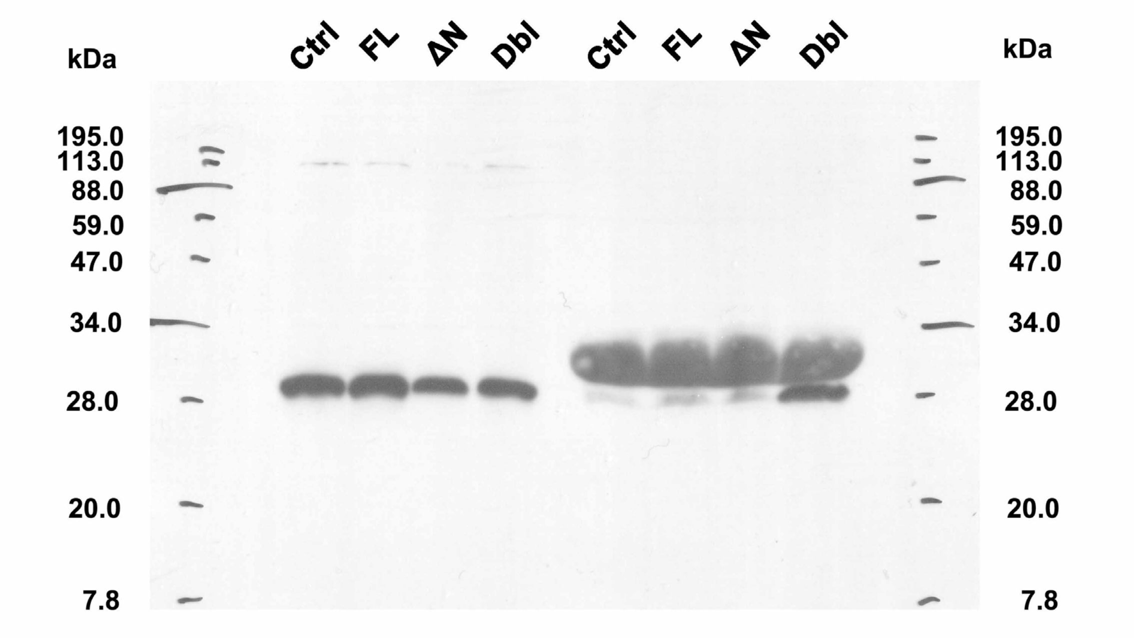

Figure S2. The full image of the western blot.

Bands of active (right) and total (left) HA-tagged RhoA in cell lysates from HEK293 cells transfected with plasmids coding genes depicted in each column were determined by immunoblotting with anti-HA antibody.

Table S1. Primer sequences

Experiments	Primer name	Sequence (5' to 3')
in situ hybridization	fRhodopsin-ISH-F	AAGTTCTCAGCGCTGGCTGCCTACATG
	fRhodopsin-ISH-R	GTGAAGATGTAGAACGCAACGCTGGCA
	fRh2-ISH-F	AATACCGCCTCGTGTGCTGCTACATCT
	fRh2-ISH-R	AGCATGAAGCCCAGCACCATGAGGATC
	fArhgef33-ISH-F	CTTCCCTGGCAACAGAGGGGCAGTCGA
	fArhgef33-ISH-R	AATAGTCAGGGTGCTCCTGCTCCGTGA
qRT-PCR	fRhodopsin-q-F	TATGGGAACCTGGTTTGCACTGTC
	fRhodopsin-q-R	AGTCACTTCCTTCTCTGCCTTCTG
	fRh2-q-F	CTCTACAACCCCATCATCTACGTG
	fRh2-q-R	CAGCAGATTGTGGTGATCATGCAG
	fArhgef33_q_F	ATGATCTCAGCCTCATCTCGAAGC
	fArhgef33_q_R	TTGTCATCCTTCTGGCAGAACCTG
	fGAPDH_q_F	ACATCAAGAGGGTAGTGAAGGCTG
	fGAPDH_q_R	CAAAGGTGGAGGAATGGCTATCAC
pCAGGSII-3xFlag-Arhgef33-FL	mArhgef33-FL-F	TCTCGAGATGGAAAAATCCAAAGCCAAGCAA
	mArhgef33-FL-R	AATCGATTCACCACCAGCCCCAAGGACCACAGAA
pCAGGSII-3xFlag-Arhgef33-delN	mArhgef33-delN-F	TGAATTCGGTTCTGTGTGAAACCTCTTTAGCTG
	mArhgef33-delN-R	AATCGATTCACCACCAGCCCCAAGGACCACAGAA
pCAGGSII-3xFlag-Arhgef33-delDH	mArhgef33-delDH-F	TGAATTCGCAATGCAATGAAGATTTGCTTATTCAG
	mArhgef33-delDH-R	AATCGATTCACCACCAGCCCCAAGGACCACAGAA
pCAGGSII-2xHA-RhoA	mRhoA-F	TGAATTCGATGGCTGCCATCAGGAAGAAACTG
	mRhoA-R	TCTCGAGTCACAAGATGAGGCACCCAGAC
